# The Sense of Agency during Continuous Action: Performance Is More Important than Action-Feedback Association

**DOI:** 10.1371/journal.pone.0125226

**Published:** 2015-04-20

**Authors:** Wen Wen, Atsushi Yamashita, Hajime Asama

**Affiliations:** Department of Precision Engineering, the University of Tokyo, Tokyo, Japan; Eberhard Karls University of Tuebingen Medical School, GERMANY

## Abstract

The sense of agency refers to the feeling that one is controlling events through one’s own behavior. This study examined how task performance and the delay of events influence one’s sense of agency during continuous action accompanied by a goal. The participants were instructed to direct a moving dot into a square as quickly as possible by pressing the left and right keys on a keyboard to control the direction in which the dot traveled. The interval between the key press and response of the dot (i.e., direction change) was manipulated to vary task difficulty. Moreover, in the assisted condition, the computer ignored participants’ erroneous commands, resulting in improved task performance but a weaker association between the participants’ commands and actual movements of the dot relative to the condition in which all of the participants’ commands were executed (i.e., self-control condition). The results showed that participants’ sense of agency increased with better performance in the assisted condition relative to the self-control condition, even though a large proportion of their commands were not executed. We concluded that, when the action-feedback association was uncertain, cognitive inference was more dominant relative to the process of comparing predicted and perceived information in the judgment of agency.

## Introduction

In present-day life, people use machines to make many tasks, such as driving cars and using computers, easier and simpler to perform. If an individual’s feeling is one of control (i.e., “I am controlling it”) while operating a machine, this helps the individual to explain and make predictions regarding feedback from the machine and take subsequent action. This subjective feeling of controlling events through one’s own behavior refers to the *sense of agency*. How does the sense of agency arise, and which factors influence this subjective feeling? These questions have been the focus of much research, in which there has been agreement that both internal motoric signals (i.e., action selection) and external cues (e.g., feedback or situational cues) contribute to the sense of agency [1].

Many previous studies have suggested that the sense of agency arises principally from a neurocognitive comparator model, which highlights the association between internal motor signals and actual sensory feedback. According to the comparator model, a predicted state is generated from one’s motor commands and compared to actual sensory feedback [2–7]. If the predicted and perceived information is matched, people will feel that the perceived event is produced via their actions and experience a sense of self-agency. If there is a mismatch, people will experience less of a sense of agency, and if the extent of the mismatch exceeds a certain sensitivity threshold, they will lose the sense of agency entirely. The comparator model is well-supported by neurocognitive studies [2,6,8] and has been used to explain delusions of control in patients with schizophrenia [9–11]. Consistent with this theory, internal motor signals have been shown to play an important role in the sense of agency. For example, people feel a greater sense of agency if they choose an action themselves (i.e., voluntary action), relative to conditions in which the action is triggered by external forces (i.e., involuntary action) [12–14]. Furthermore, the fluency of action selection has been reported to influence the feeling of self-agency [12], revealing the important role of internal premotor signals in the sense of agency, as the action selection process is considered to influence the strength of internal motor signals.

However, recent studies have demonstrated cases in which consistency between internal motor signals and actual sensory feedback is unnecessary for the development of a sense of agency. For example, Wegner and colleagues developed an interesting paradigm that induced a false sense of agency [15]. In their experiment, the participants watched themselves in a mirror without moving, while a paired participant, the “helper,” stood immediately behind the participant, with his or her arms extended outward, and performed a series of movements. In the condition in which participants heard instructions previewing each movement, they reported a (false) sense of control over the helpers’ hands. This phenomenon of vicarious agency emphasized the role of external cues in the sense of agency. To account for external factors in the sense of agency, Synofzik and colleagues proposed a two-step model of the sense of agency, which included a perceptual level, involving the feeling of agency, and an explicit conceptual level, involving the judgment of agency [16]. Synofzik et al. suggested that the sense of agency is a combination of these two types of process. In ambiguous situations in particular, external cues could play a more important role in the judgment of agency [16]. In a recent electrophysiology study, sensory attenuation of an early potential (N1) was observed for a learned action-feedback association, whereas attenuation of a later potential (P3a) was observed for agency judgment [17]. The authors suggested that the detection of unpredicted information was reflected in early sensory attenuation processes, but the judgment of agency was drawn from more cognitive mechanisms [17]. A similar study found that associations between different components of event-related potentials and participants’ agency judgments differed according to the reliability of the association between action and feedback [18]. These findings are also consistent with the cue integration theory of agency, which suggests that the reliability of both internal and external cues determines the extent to which they contribute to the sense of agency [1]. In the present study, we investigated the dominance of different cues involved in the judgment of agency in conditions that differed with respect to the reliability of the action-feedback association. We hypothesized that if the congruence between predicted and actual sensory information was less reliable, experience of a sense of agency would be greatly influenced by task performance, which is an external cue.

The consistency between prediction and subsequent feedback has been shown to influence the judgment of agency in numerous studies [17,19–23]. For example, people experienced a stronger sense of agency when their actions caused effects that were congruent with prior knowledge, relative to a condition in which effects were incongruent with prior knowledge [19,20,24]. If the representation of an event that followed an action was activated via priming, the sense of agency regarding the action was enhanced, regardless of whether the prime was subliminal [21,22] or supraliminal [25]. Furthermore, when the prediction matched actual sensory feedback to a greater extent, this enhanced the “intentional binding” effect [26], a phenomenon in which the perceived duration of the interval between an action and its corresponding effect is compressed when the feeling of agency is experienced, indicating that the first-order, pre-reflective experience of agency was promoted [23]. These studies used a common paradigm in which participants performed a single action and waited for corresponding feedback. Under these conditions, predicted and actual sensory feedback would be easy to compare.

However, in many situations in which people are required to perform continuous actions and simultaneously receive continuous feedback (e.g., driving a car), it is difficult to predict accurate feedback for every single action. Rather, people could predict the flow of feedback ambiguously and make less accurate comparisons between the predicted state and actual sensory feedback relative to conditions involving single-action feedback. Moreover, if the actions are accompanied by a goal, a high-level cognitive inference could be drawn: if people are able to take control, they should achieve the goal satisfactorily. The reverse inference (which we refer to as “goal-directed inference” in the following content) could play a role in the judgment of agency. That is, people would believe themselves to be in control when they achieve positive results from a task [27]. Metcalfe and Greene further examined this reconstructive process with respect to the judgment of agency, and found that people’s judgment of self-agency was correlated with their task performance, even when they were explicitly aware that the enhancement of their performance was largely the result of external factors [28]. We propose that, as the comparison between continuous action and feedback is difficult, people rely heavily on other external factors, such as task performance, in the judgment of agency during continuous action. That is, goal-directed inference plays a critical role in the generation of a sense of agency when people are engaged in continuous action.

In summary, we suggest that both the action-feedback association and goal-directed inference influence the judgment of agency in a task involving continuous action accompanied by a goal, and when the former is less reliable, the latter plays a dominant role. To our knowledge, no research has been conducted to examine the dominance of the two types of process in the judgment of agency. To examine this issue, we propose a novel paradigm, in which people control an object on a computer screen, with the aim of directing the object to reach a goal as quickly as possible. In the background, the program ignores erroneous commands in some instances, which raises performance levels but simultaneously weakens the participant’s control (because some of the participants’ commands are not executed). If the action-feedback association is dominant in the judgment of agency, people will feel less of a sense of agency when their commands are only partially executed. Conversely, if the goal-directed inference is dominant, people will feel a greater sense of agency, as their performance level improves, even though their commands are not fully executed.

## Method

In the experiment, participants controlled the direction of a moving dot and aimed to position it at a target location as quickly as possible. The difficulty of the action-feedback association was varied by manipulating the delay in the dot’s response, as suggested by the comparator model, in which sensory feedback deviates from the predicted state when there is a delay [7]. In half of the trials, the program was designed to ignore erroneous commands that caused the dot to deviate from the direction of the target location (the assisted condition). In the other half of the trials, the program executed all of the participants’ commands (the self-control condition). Following each trial, participants rated their sense of agency. Task performance was measured via two indices, the average time required and number of key presses used in each trial. Judgments of control and task performance were compared between the assisted and self-control conditions. We also conducted a multivariate analysis to examine goal-directed inference by assessing synchronized changes in task performance and the sense of agency.

### Participants

A total of 17 students with normal or corrected-to-normal visual acuity participated in the experiment. Their mean age was 25.5 years (*SD* = 2.8, range 23–32). The experiment was conducted with the approval of the ethics committee of the Faculty of Engineering at the University of Tokyo, and written informed consent was obtained from all participants.

### Stimuli and Task

**Fig 1:**
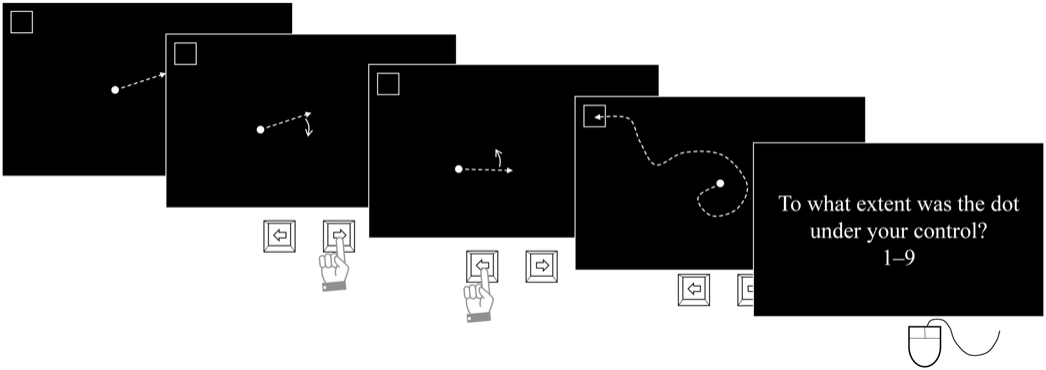
The flow of each trial of the experimental task. Arrows with broken lines indicate the direction in which the dot moved. Participants were instructed to direct the moving dot into the square as quickly as possible by pressing the left or right key to turn the moving dot clockwise or counterclockwise, respectively. After moving the dot to the destination, they used a mouse to rate the extent to which they felt that the dot was under their control, using a 9-point scale.

In each trial of the experimental task (Fig 1), a 6-mm white dot appeared in the center of the 597 mm × 336 mm (width × height) black screen and moved at a frequency of 124 mm/s in a fixed direction, if participants did not press the left or right key. The original direction of the dot was random (differed between trials). A 31-mm black square with a white border appeared at one of the four corners of the screen; this was the destination for the duration of the trial. Participants were instructed to press the left or right key on the keyboard to change the direction of the moving dot and direct the dot into the square as quickly as possible. The direction of the dot turned 10° clockwise with a right key press and 10° counterclockwise with a left key press. If participants held the left or right key down, the direction of the moving dot would turn at a frequency of 190°/s in the first second and 330°/s subsequent to this. Participants were informed that the keys should be held down to change the direction of the dot rapidly and pressed briefly to make fine adjustments. If the dot moved outside of the screen, it would appear at the opposite side of the border and continue to move in the same direction. Once the dot appeared at the target location, participants rated the extent to which they felt that the dot was under their control on a 9-point scale (1 = not at all; 9 = a lot) by using a mouse to click radio buttons.

**Fig 2:**
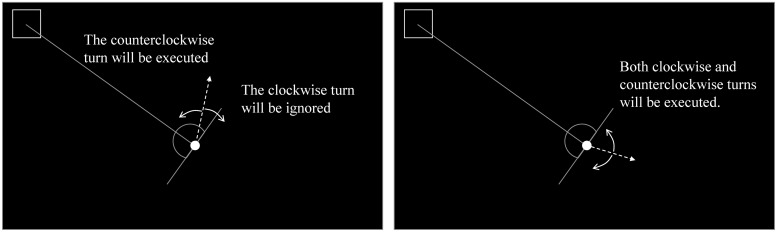
An example of the assisted condition. In the assisted condition, when the angle between the direction of the dot and the target location was less than 90° (the left figure; i.e., when the direction of the dot was within the range shown with the semicircle), commands that caused the dot to move away from the target location (clockwise turn in the left figure) were ignored, while commands that caused the dot to move toward the target location were executed (counterclockwise turn in the left figure). When the angle between the direction of the dot and target location was at least 90° (the right figure), all commands were executed.

There were three possible delays (100, 400, and 700 ms) between the participant’s key press and the dot’s response (i.e., direction change). The delay was consistent within each trial and varied randomly between trials. In half of the trials, when the angle between the direction of the dot and the target location was less than 90°, commands that caused the dot to move away from the target location were ignored (the assisted condition, Fig 2). In other trials, all of the participants’ commands were executed (the self-control condition). The two types of trial were combined in random order.

### Procedure

Participants were tested individually, seated on a chair positioned 50 cm away from a 27-inch LCD monitor with a resolution of 1,920 × 1,080 pixels. Having received an explanation regarding the requirements of the experimental task, they practiced 3 times without delay and 3 times with a delay of 300 ms. Participants were not notified about the computer assistance; rather, they were instructed that the response of the dot would sometimes be delayed or out of their control. The delay condition was practiced repeatedly until the participants were able to complete a trial within 10 s. Following practice, each participant completed 60 trials comprising 10 trials involving each delay duration (100, 400, and 700 ms) in each assistance condition (assisted vs. self-control), in random order. Upon completion of all trials, participants provided an oral report detailing the number of times they had felt that an operation had been invalid, and whether they had noticed a specific rule when their commands did not appear to have been executed. The experiment lasted for 30 min, on average.

## Results

### The Rating of Control

**Fig 3:**
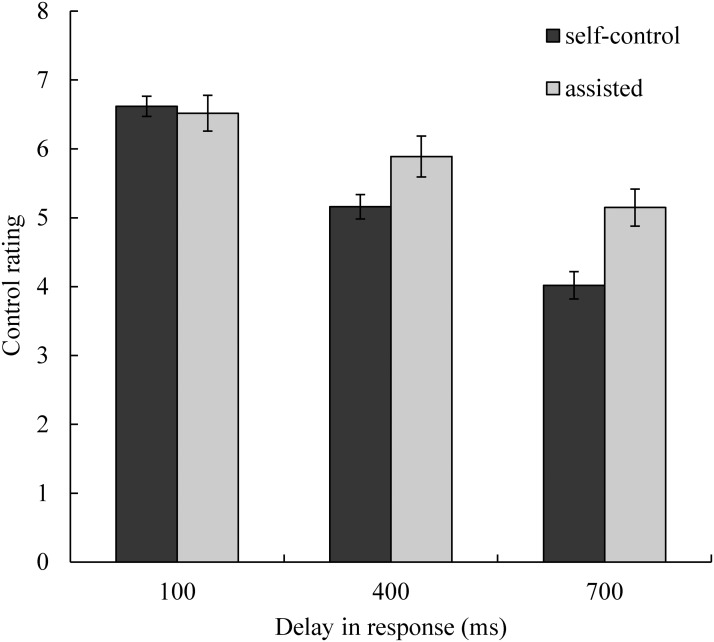
Mean control ratings in each condition. Error bars represent standard errors. The rating scores decreased significantly with incremental delays in response. The differences between the assisted and self-control conditions were significant in the 400 ms and 700 ms conditions but non-significant in the 100 ms condition.

The average ratings and standard errors for each condition are shown in Fig 3. We conducted a 3 (delay of 100, 400 or 700 ms) × 2 (assisted control or self-control) repeated measures ANOVA to compare rating scores. Unsurprisingly, the main effect of delay was significant (*F*(2, 32) = 84.57, *p* <. 01, η_p_^2^ = 0.84). The longer the delay in the response of the dot, the lower control was rated. Post-hoc comparisons revealed significant differences between the three delay conditions. Tukey’s HSD test indicated that the 100 ms and 400 ms conditions and the 400 ms and 700 ms conditions differed significantly (*p*s *<*. *01)*. More importantly, the main effect of assistance was significant (*F*(1, 16) = 8.92, *p* <. 01, η_p_^2^ = 0.36), indicating that participants reported greater control in the assisted condition relative to the self-control condition. Moreover, the interaction between delay and assistance was significant (*F*(2, 32) = 8.87, *p* < .01, η_p_^2^ = 0.36). Post-hoc comparisons revealed that rating scores did not differ between the control and assisted conditions when the delay was 100 ms (Tukey’s HSD test: *p* = .997), but differed significantly when the delays were 400 ms or 700 ms (Tukey’s HSD test: *p*s < .05 and < .01, respectively).

### Time Required in Each Trial

**Fig 4:**
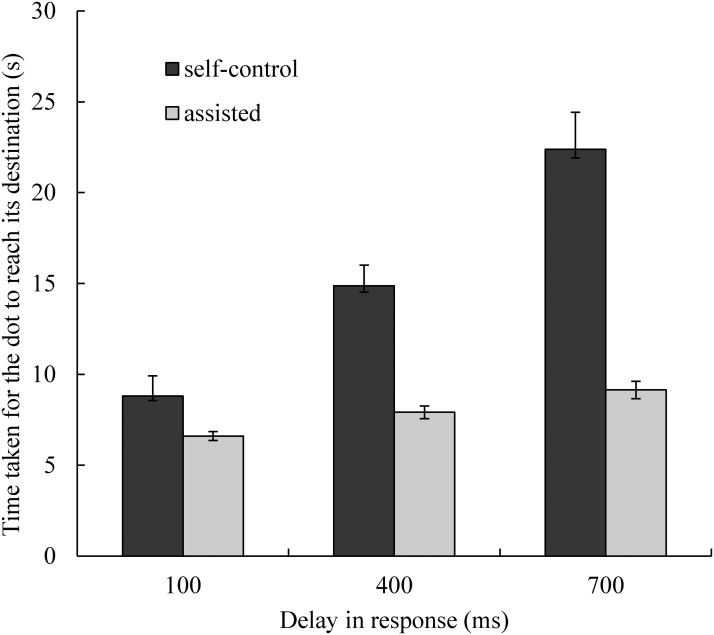
Average time required in each trial (from the presentation of the dot until arrival at the target location). Error bars represent standard errors. The time required to complete each trial increased significantly with incremental delays in response. The time required did not differ between the self-control and assisted conditions when the delay was 100 ms, but differed significantly when the delay was 400 ms or 700 ms.

Because participants were instructed to direct the moving dot into the box as quickly as possible, the average time from the appearance of the dot to the arrival at the target location in each trial served as a direct index of task performance (the shorter the time required, the better the performance; Fig 4). We conducted a 3 (delay) × 2 (assistance) repeated measures ANOVA to compare the time required between conditions. The main effect of delay was significant (*F*(2, 32) = 71.93, *p* < .01, η_p_^2^ = 0.82). Post-hoc comparisons showed that participants required more time in the conditions with longer delays in the dot’s response. Tukey’s HSD test revealed significant differences between the 100 ms and 400 ms conditions and the 400 ms and 700 ms conditions (*p*s < .01), indicating that difficulty increased with longer delays; this was unsurprising. The main effect of assistance was also significant (*F*(1, 16) = 45.52, *p* < .01, η_p_^2^ = 0.74). The time required in the assisted condition was shorter than that required in the self-control condition, indicating that computer assistance improved performance. Moreover, the interaction between delay and assistance was significant (*F*(2, 32) = 31.59, *p* < .01, η_p_^2^ = 0.66), suggesting that the enhancing effect of assistance in task performance varied according to task difficulty. Post-hoc comparisons showed that the time required did not differ between the self-control and assisted conditions when the delay was 100 ms (Tukey’s HSD test: *p* = .25), but differed significantly when the delay was 400 ms or 700 ms (Tukey’s HSD test: *p*s < .01). This was unsurprising, because participants’ performance probably contained more erroneous operations when the delay in response was longer; therefore, computer assistance played a more important role in improving task performance.

### Number of Key Presses in Each Trial

**Fig 5:**
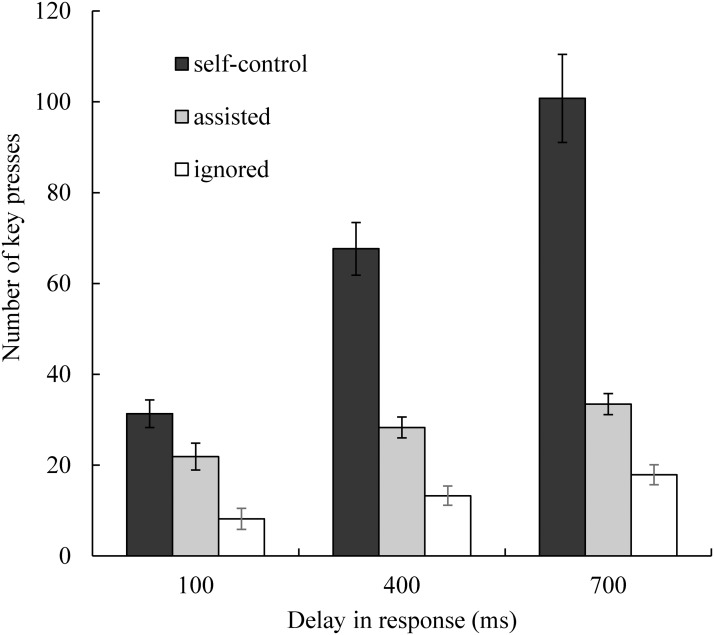
The number of times participants pressed the left or right key and the number of ignored key presses in each trial. Error bars represent standard errors. The participants pressed the keys more often when the delay in response was longer. The number of times participants pressed the keys did not differ significantly between the assisted and self-control conditions when the delay was 100 ms, but when the delay increased to 400 ms or 700 ms, they pressed the keys fewer times in the assisted condition relative to the self-control condition. The proportion of ignored operations was greater in the 400 ms and 700 ms conditions relative to the 100 ms condition.

The number of times participants pressed the left or right keys served as another index of task performance (fewer key presses indicated better performance). This index reflected the number of erroneous actions performed and was probably related to the time required to complete each trial. The total number of key presses (including ignored presses) and the number of ignored key presses are depicted in Fig 5. Again, a repeated measures ANOVA (delay × assistance) was conducted to compare total numbers of key presses. The main effect of delay was significant (*F*(2, 32) = 54.39, *p* < .01, η_p_^2^ = 0.77). Post-hoc comparisons indicated that participants pressed the keys more often when the task was more difficult. Tukey’s HSD test revealed significant differences between the 100 ms and 400 ms conditions and the 400 ms and 700 ms conditions (*p*s < .01). The main effect of assistance was significant (*F*(1, 16) = 54.01, *p* < .01, η_p_^2^ = 0.77). The participants pressed the keys fewer times in the assisted condition relative to the self-control condition. The interaction between the two factors was also significant (*F*(2, 32) = 31.36, *p* < .01, η_p_^2^ = 0.66). Post-hoc comparisons revealed that the number of times that participants pressed the keys did not differ significantly between the assisted and self-control conditions when the delay was 100 ms (Tukey’s HSD test: *p* = .46), but when the delay increased to 400 or 700 ms, keys were pressed fewer times (*p*s < .01) in the assisted condition relative to the self-control condition.

To compare the proportions of ignored and total key presses, we conducted a single-factor (delay) repeated measures ANOVA. Angular transformation was applied in the analysis (one participant’s ignored key presses were lost due to a technical problem). The main effect of delay (*F*(2, 30) = 7.82, *p* < .01, η_p_^2^ = 0.34) was significant. The post-hoc test showed that the proportions of ignored operations were greater with 400 ms and 700 ms delays relative to a 100 ms delay (Tukey’s HSD test: *p*s < .05 and < .01 respectively). However, the proportions of ignored operations did not differ significantly between the conditions with 400 ms and 700 ms delays (Tukey’s HSD test: *p* = .38).

### Frequency of Key Presses

**Fig 6:**
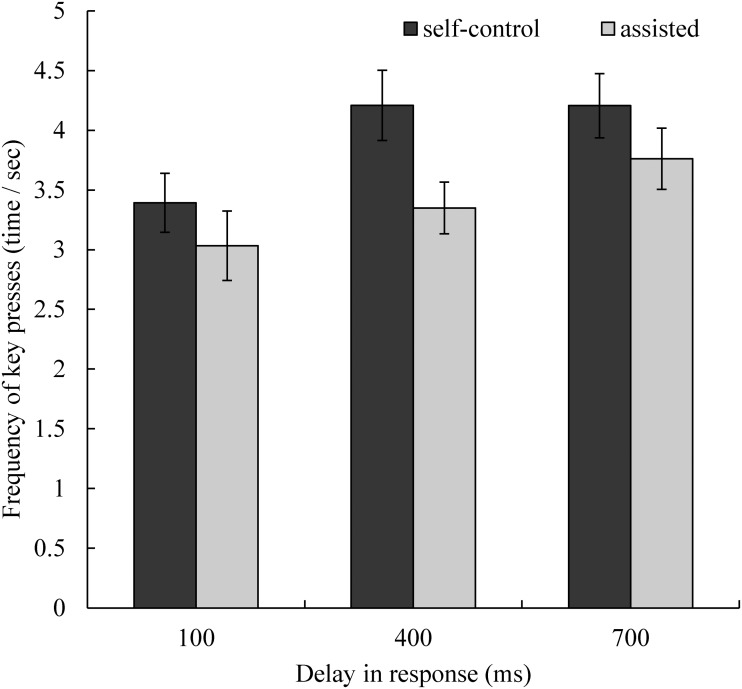
Frequency of key presses in each condition. Error bars represent standard errors. We believe that the frequency of key presses reflects the extent of feelings of control. The higher the frequency of key presses, the less control is experienced. The participants pressed the keys more frequently in the 400 ms and 700 ms conditions relative to the 100 ms condition, while the difference between the two longer delay conditions was non-significant. Participants pressed the keys less frequently in the assisted condition relative to the self-control condition. The interaction between delay in response and assistance was non-significant.

We calculated the frequency of key presses by dividing the number of key presses by the time required in each trial (Fig 6), to determine whether the participants pressed the keys more frequently (in a time unit) when the task was more difficult. Because the frequency of key presses was calculated using two indices of task performance, it may not have been an appropriate means of performance measurement. Rather, it may have reflected participants’ behavioral patterns, and we wished to determine whether it was correlated with the sense of agency. We conducted a repeated measures ANOVA (delay × assistance). The main effect of delay was significant (*F*(2, 32) = 11.12, *p* < .01, η_p_^2^ = 0.41). Post-hoc comparisons showed that participants pressed the keys more frequently with 400 ms and 700 ms delays relative to a 100 ms delay (Tukey’s HSD test: *p*s < .01 and < .01 respectively), while the difference between conditions with 400 ms and 700 ms delays was non-significant (Tukey’s HSD test: *p* = .46). The main effect of assistance was also significant (*F*(1, 16) = 11.79, *p* < .01, η_p_^2^ = 0.42), indicating that participants pressed the keys less frequently in the assisted condition relative to the self-control condition. The interaction between delay and assistance was non-significant (*F*(2, 32) = 1.13, *n*.*s*., η_p_^2^ = 0.07).

### Correlations

We calculated correlations between delay, assistance (assisted condition = 1; self-control condition = 0), time required, number of key presses, frequency of key presses, and agency rating (Table 1) for each participant. We conducted two-tailed one-sample *t* tests and found that all of the correlations shown in Table 1 differed significantly from. 00 (Fisher’s *z* transformation was applied for the analyses, *p*s < .01).

**Table 1:** Average correlations and standard deviations.

		3	4	5	6
Independent Variables	1. Delay	.34 (.07)	.29 (.06)	.16 (.11)	-.38 (.12)
	2. Assistance	-.36 (.07)	-.33 (.09)	-.13 (.17)	.14 (.19)
Task Performance	3. Time Required		.84 (.08)	.27 (.14)	-.52 (.18)
	4. Number of Key Presses			.65 (.10)	-.54 (.16)
	5. Frequency of Key Presses				-.35 (.15)
Sense of Agency	6. Control Rating				

Correlations were analyzed using Fisher’s *z* transformation and compared to. 00 using two-tailed *t* tests. All of the correlations shown differed significantly from. 00 (Fisher’s *z* transformation was applied for the analyses, *p*s < .01).

### Multivariate Analysis

The main purpose of the multivariate analysis was to examine goal-directed inference. Because both task performance and sense of agency were dependent variables, it was impossible to examine causality between them. However, multivariate analysis allowed us to estimate the extent to which task performance influenced the sense of agency and compare it with the influence exerted by action-feedback association. In the multivariate analysis, the direct path from delay to the sense of agency reflected the influence of action-feedback association on the sense of agency, while the indirect path via task performance reflected goal-directed inference.

The two independent variables, delay and association, were used to manipulate the difficulty of the action-feedback association. Specifically, feedback was easier to associate with correspondent actions when the delay was shorter or an association was absent. Association was also used to improve task performance. Task performance was assessed according to two indices: the time required to complete each trial and the number of key presses required to guide the dot to the destination in each trial. The two indices were highly correlated (*r* = .84). Sense of agency was examined using subjective rating scores.

**Fig 7:**
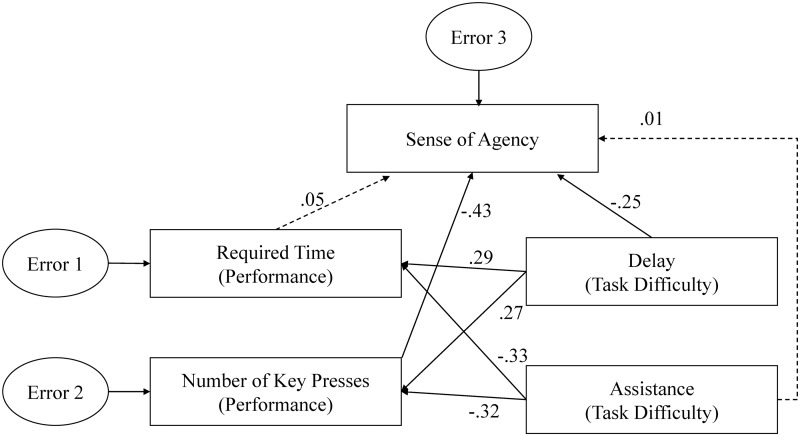
The full structural equation model (i.e. path model) of sense of agency and its predictors. The paths with significant coefficients are represented by solid lines and the paths with non-significant coefficients by broken lines. The model indicated that sense of agency was influenced by delay via both direct and indirect routes but was influenced by assistance indirectly via task performance.

We performed structural equation modeling using IBM SPSS Amos 22 (Fig 7). In the model, sense of agency was influenced by delay and assistance via direct and indirect paths. In the indirect path, delay and assistance influenced the indices of task performance, and task performance then influenced sense of agency. The direct paths from delay and association to the sense of agency reflected the influence of the action-feedback association on the sense of agency, and the indirect paths from delay and association to the sense of agency via indices of task performance (which was our focus), reflected the influence of goal-directed inference on the sense of agency.

The standardized coefficients for all paths are depicted in Fig 7, and the fit parameters are shown in Table 2. Non-significant paths are shown as broken lines, and significant paths are shown as solid lines. According to the results of the analysis, the path from delay to sense of agency was statistically significant (-.25), but the path from assistance to sense of agency was non-significant (.01). In contrast, the indirect path from assistance to sense of agency via number of key presses was significant. In summary, sense of agency appeared to be influenced by delay via both direct and indirect routes but was influenced by assistance indirectly via task performance.

**Table 2:** Fit indices for the full and simplified structural equation models of the sense of agency.

	χ^2^ / df	GFI	AGFI	NFI	CFI	RMSEA
Full model	484.350	.803	-.478	.435	.433	.689
Simplified model	.000	1.000	1.000	1.000	1.000	.000
Standard of good fit	<2.0	≥.90	≥.90	≥.90	≥.90	≤.08

The standard of good fit is provided in the last row. The simplified model fit the data very well.

**Fig 8:**
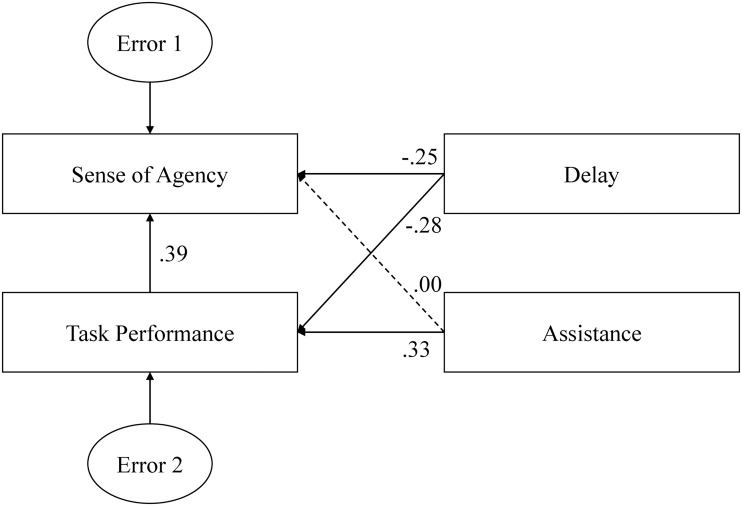
The simplified structural equation model (i.e., path model) of the sense of agency and its predictors. The paths with significant coefficients are represented by solid lines and the paths with non-significant coefficients by broken lines. The two original indices of task performance were combined to form another index entitled task performance. The model clearly indicated that delay influenced the sense of agency via both direct and indirect paths, while assistance only influenced the sense of agency via an indirect path.

To compare the direct and indirect paths more closely, we combined the two indices of task performance to form one index entitled task performance. To do this, we calculated standard scores (*z*-values) for the original indices (subtracting the mean from the raw data and dividing the result by the standard deviation). The index of performance was the additive inversed averages of standardized time required and number of key presses. The simplified model and results of multivariate analysis are shown in Fig 8, and the fit parameters are shown in Table 2. The simplified model fit the data very well and clearly showed that assistance influenced the sense of agency indirectly via task performance, and delay influenced the sense of agency directly. Specifically, the coefficients for the direct and indirect paths from assistance to sense of agency were. 00 and. 13 (i.e., multiplication of the coefficients for the paths from assistance to task performance and task performance to sense of agency), respectively. The coefficients for direct and indirect paths from delay to sense of agency were-.25 and-.11(i.e., multiplication of the coefficients of the paths from delay to task performance and task performance to sense of agency), respectively.

## Discussion

The purpose of the present study was to examine the dominance of two types of process influencing judgment of self-agency during continuous action. The first type of process refers to the comparison between predicted and perceived information. The second type of process refers to a higher-level cognitive inference—we named this goal-directed inference—that if the outcome of people’s actions meets their expectations with respect to the goal (e.g., good task performance), they infer that events are under their control. The results of the present study clearly demonstrated that goal-directed inference influenced judgment of self-agency to a greater extent relative to the comparison between predicted and observed movement of the dot when the response of the dot was delayed (≥ 400 ms). Specifically, the participants’ sense of agency was enhanced when their performance improved through computer assistance, even though a large proportion of their commands (average proportions of ignored commands were 31.5%, 43.5%, and 50.4% with delays of 100 ms, 400 ms, and 700 ms, respectively) were not executed.

The process involved in comparing predicted and perceived information has been shown to play a critical role in the generation and judgment of the sense of agency [12,13]. However, the role of goal-directed inference has been discussed in fewer studies. To our knowledge, only two studies have indicated that task performance plays an important role in the judgment of self-agency [28,29], and the present study was the first to examine the dominance of the two types of process, goal-directed inference and the comparison between predicted and perceived states, during this judgment. We discuss the findings of the present study individually below.

First, consistent with findings from a number of previous studies, we found that difficulty associating an action with its effect significantly influenced sense of agency. When the delay in the response of the dot increased, associating one’s command with the corresponding change in the dot’s direction became more difficult. As a result, participants’ control ratings were lower (Fig 3). Previous studies in which the temporal interval between action and feedback was adjusted also demonstrated that longer intervals between action and feedback resulted in weaker sense of agency [14,19,20,30]. One might wonder whether poor task performance was solely responsible for the influence of delay on sense of agency in conditions with longer delays. Based on our results, we believe that this is unlikely. For example, in the 400 ms condition with computer assistance, performance levels were similar to (or better than) those of the 100 ms condition without computer assistance (see Figs 4 and 5). However, control rating scores in the 400 ms condition with computer assistance were significantly lower relative to those observed in the 100 ms condition without computer assistance (Tukey’s HSD test: *p* < .05). Furthermore, according to the simplified path model (Fig 8), the coefficient for the direct path from delay to sense of agency was larger relative to that of the indirect path via task performance.

The association between action and effect in the mechanism underlying sense of agency has been discussed in numerous studies. Many previous studies have used a single action-feedback paradigm to examine factors influencing this association and reported a number of important findings. For example, voluntary actions are considered to produce stronger motor signals, relative to involuntary actions, and result in a stronger sense of agency [12–14], as stronger motor signals enhance the action-feedback association. Furthermore, many studies have reported that, if the representation of the effect has been learned [19,20,24] or activated prior to action (e.g., by the presentation of a prime) [21,22], the sense of agency toward the action would be stronger relative to that observed with unpredicted or inactivated effects. In addition to these factors, many other external cues related to action and effects, such as priming an action [31,32], the emotion involved in outcomes [33], and the quantity of outcomes [20], also influence sense of agency. All of these manipulations are believed to influence the association between action and effect. Furthermore, a well-known phenomenon, the intentional binding effect [26], which involves a subjective attraction between action and effect, also reflects the implicit association process that occurs.

Second, the most important finding of the present study was that during continuous action with a goal, goal-directed inference played an important role in the sense of agency, and in some cases, it could even overwrite the processes involved in the action-feedback association. In our experiment, the computer ignored a large proportion of participants’ erroneous commands (see Fig 5), thereby improving participants’ performance levels greatly (see Figs 4 and 5). That is, although participants’ performance improved, their actual control was reduced by approximately 42% (averaged across all delay conditions). However, only one participant noticed that the dot did not respond to a large proportion of commands when it was moving directly toward the destination, and all of the participants reported higher levels of control in the assisted condition relative to the self-control condition. To estimate the influence of goal-directed inference, we conducted a multivariate analysis and found that the aggregate coefficients of the indirect paths from delay and association to the sense of agency via indices of task performance, which reflected goal-directed inference, were larger relative to those of the direct paths (Fig 7,. 28 vs. 26). In summary, during the judgment of agency, goal-directed inference seemed to play a more important role in the judgment of agency relative to the action-feedback association.

Previous studies have revealed the important role played by goal-directed inference in the judgment of agency [28,29]. Metcalfe and Greene asked participants to play a simple PC game and varied the difficulty of the game [28]. In their experiments, the participants used a mouse to move a box across a horizontal track on a computer screen, catching Xs and avoiding Os as the track traveled downward. The turbulence of the mouse, scroll speed, and density of the targets (i.e., Xs) were manipulated throughout the experiments. Metcalfe and Greene found that participants’ judgment of self-agency was greatly influenced by their performance in the game, even when their performance benefited from more lenient rules (i.e., the magic condition, in which hits were counted when the box neared the Xs but did not catch them). However, in this research, better task performance was always accompanied by less disruption to the control of the object (i.e., a less turbulent mouse); therefore, it was not possible to examine the dominance of goal-directed inference in performance, or the association between action and feedback. In the present study, we not only demonstrated the strong influence of goal-directed inference on the sense of agency, we also found that this influence could overwrite the influence of the association between action and effect.

Synofzik and colleagues proposed a two-step model to describe the generation of a sense of agency [16]. In the first step, the basic nonconceptual feeling of agency is generated via motor signals and action-related perceptual cues. In the second step, a propositional representation of the sense of agency is formed with the nonconceptual feeling of agency, conceptual attitudes, and contextual cues [16]. Similar to the concept of the two-step model, recent studies have also suggested that judgment of agency is a reconstructive process reflecting higher-level cognitive processes [17,34,35], and both predictive and inferential processes contribute to the sense of agency [36]. Our results also support this assertion. Sense of agency was influenced by both task performance and the association between action and feedback (see the simplified model in Fig 8, in which both direct and indirect path coefficients were significant). Furthermore, our results suggest that, in the condition in which it was difficult to associate actions with their consequences, task performance may have attributed to the sense of agency to a much greater extent relative to the action-feedback association. In the two-step model of agency [16], Synofzik and colleagues suggested that the more ambiguous agency is, the more important the process in the second step becomes. Our results provided evidence for this hypothesis, in that the influence of task performance on judgment of agency was significant in the conditions involving longer delay, in which the participants probably experienced greater difficulty in specifying agency with respect to the dot’s movement, but was non-significant in the condition involving a 100 ms delay, in which participants found it easier to compare their predictions of the dot’s movements with actual observed movements.

Moore and Fletcher have proposed a theory of cue integration in the sense of agency [1]. In this framework, both internal and external cues are thought to influence the sense of agency, and the extent to which they contribute to the sense of agency is determined by their reliability. Our results were consistent with the cue integration theory. Specifically, when the delay between key presses and the response of the moving dot was very short (i.e., 100 ms), the sensorimotor information was relatively reliable (i.e., if the participant pressed the right key twice, he or she would expect a 20° clockwise turn of the dot) and contributed to the sense of agency to a large extent. In this condition, the goal-directed inference did not play significant role in the sense of agency. In contrast, when the delay was longer, the sensorimotor information became less reliable; as a result, the influence of the goal-directed inference became dominant and played significant role in the production of a sense of agency.

In the present study, we used delay in the dot’s response to vary the reliability of the action-feedback association. For healthy individuals, long delays between motor commands and their consequences tend to be uncommon in daily life, due to advances in technology. However, there are cases in which the action-feedback association may be ambiguous in daily life. For example, if a number of individuals are collaborating with each other and handling the same object, or one’s single command could cause more than one consequence, the action-feedback association could be uncertain. In summary, we believe that the goal-directed inference plays a significant role when the action-feedback association is uncertain but does not do so when the association is reliable.

In light of our findings, we suggest that the processes involved in the judgment of agency probably differ between conditions involving control of an external object with a goal and those without a goal. In operation without a goal, the sense of agency could arise as a result of bottom-up processes (i.e., comparing predicted and perceived information). Conversely, if the operation includes a goal, sense of agency may serve as a belief (e.g., “I can control this object”), whereby people select actions and direct their attention toward achievement of the goal rather than checking every state of the object. Differences in attentional level probably resulted in varying dominance with respect to effect-feedback associations and goal-directed inference. Unfortunately, we did not ask the participants why they experienced a greater sense of agency in the assisted condition relative to the baseline condition. The reason why goal-directed inference was dominant in judgment of the sense of agency should be explored in future research.

## Conclusion

The present study examined the influence of task performance and the action-feedback association on the sense of agency during continuous actions accompanied by a goal. The results suggest that both factors influenced the sense of agency, but based on task performance, the goal-directed inference played a dominant role in the judgment of sense of agency when the action-feedback association was uncertain. The participants felt strong sense of agency when their task performance improved via computer assistance, even though a large proportion of their commands were not executed. Furthermore, a recent study has suggested that automation technology might weaken the sense of agency [37]. The present study offers inspiration, in that if automation technology could improve task performance (without the operator’s awareness), it would probably enhance the sense of agency.

## Supporting Information

S1 File The raw data of the experiment. (XLS)
